# Tricks of Nature From the Ancient Earth and Early Mars: Chemical Gardens Generate Biomorphs With High Preservation Potential

**DOI:** 10.1111/gbi.70045

**Published:** 2026-04-21

**Authors:** Solomon Hirsch, Sean McMahon, Jens Najorka, Mark A. Sephton

**Affiliations:** ^1^ Department of Earth Science and Engineering Imperial College London London UK; ^2^ UK Centre for Astrobiology, School of Physics and Astronomy University of Edinburgh Edinburgh UK; ^3^ School of GeoSciences University of Edinburgh Edinburgh UK; ^4^ Natural History Museum London UK

## Abstract

Observations of morphology are commonly used to evaluate the biogenicity of terrestrial microfossils and could constitute a crucial line of evidence for extraterrestrial life‐detection missions in the future. However, evaluating the origin of morphological features in the rock record can be problematic because naturally occurring abiotic structures can resemble biological morphologies, which may lead to false‐positive detections of fossilised life. Iron‐mineralised chemical gardens have been highlighted as potentially confounding abiotic structures because of their morphological and chemical resemblance to biomineralised filaments. Despite this, the potential for chemical garden structures to be preserved in the fossil record has not been thoroughly investigated. Here, we subjected abiotic iron‐mineralised chemical garden structures to artificial maturation using hydrous pyrolysis, in order to evaluate their preservation potential. We found that these abiotic filaments were relatively resistant to degradation caused by maturation when compared with analogous biological material. Additionally, the transformation of ferrihydrite to crystalline iron oxides was found to be relatively inhibited, likely because of the influence of silica. These findings highlight the need for fossilised filamentous material to be distinguished from chemical garden structures before a biological origin can be confidently attributed, particularly when observed in significantly altered rocks.

## 1 Introduction

The morphology of lithified microstructures is routinely used as a primary line of evidence for past life (e.g., Knoll 1992; Schopf 1993). However, the potential for abiotic reactions to form structures resembling biological materials (biomorphs) means that abiotic mechanisms must be carefully excluded to confidently establish a biotic origin (Brasier et al. 2002; García Ruiz et al. 2002; Javaux 2019). ‘Chemical garden’ reactions of metal salts in alkaline carbonate or silicate solutions have been identified as a possible source of such biomorphs (McMahon 2019). Chemical garden reactions precipitate a broad range of structures by nucleation onto a gelatinous membrane formed around metal salt solutions, typically with repeated osmotic inflow and rupture (Barge et al. 2015; Cartwright et al. 2002; Kotopoulou et al. 2021). Specifically, iron‐mineralised chemical gardens prepared from the reaction of iron salts in sodium silicate solution can mimic the morphologies of the earliest purported microfossils (McMahon 2019). Iron‐mineralised chemical garden material is dominated by tubular (hollow) biomorphic filaments, which exhibit both straight and curved morphologies with frequent branches. These biomorphs display morphological features previously considered as evidence for biogenicity, such as circular cross‐sections, consistent diameters, sinuous trajectories, branching, and anastomosis (McMahon 2019).

The formation of iron‐mineralised chemical gardens has not been directly observed in nature but has been predicted because iron and silica abound in some far‐from‐equilibrium geological settings, especially in hydrothermal systems (García‐Ruiz et al. 2017; Johannessen et al. 2020; McMahon 2019; McMahon et al. 2021). Iron‐mineralised structures ranging from macroscopic sulfide ore deposits to microscopic subseafloor filaments and even corrosion rusticles on ships have been interpreted as possible chemical gardens (Barge et al. 2015; McMahon et al. 2021; Silva‐Bedoya et al. 2021). As a result, the morphology of iron‐mineralised tubes and filaments that resemble microbial structures cannot be considered conclusive independent evidence for biogenicity if their formation can instead be explained by chemical garden reactions (Javaux 2019; McMahon et al. 2021; McMahon and Cosmidis 2021). In general, further independent lines of evidence such as chemical or isotopic analyses should be used to confirm the biogenicity of structures where an abiotic origin cannot be excluded based on morphology alone (Neveu et al. 2018; Westall et al. 2021). However, many of the most ancient (and typically most contentious) microfossils exhibit limited evidence for biogenicity beyond morphology (e.g., Dodd et al. 2017; Rasmussen 2000). Some of these ancient microfossils are compositionally and morphologically similar to iron‐mineralised chemical garden material, highlighting the importance of understanding the formation and preservation potential of both the biological and abiotic structures.

Consideration of the potential for abiotic biomorph formation and preservation could also be paramount for astrobiological studies searching for biosignatures of extraterrestrial life. Mars is a key target for biosignature‐search missions, primarily due to the evidence for liquid water during the early history of the planet, which provided potentially habitable surface conditions (Vago et al. 2017; Westall et al. 2015). The aqueous period on the Martian surface is widely considered to have occurred early and lasted relatively briefly (Wordsworth 2016). By comparison to the evolution of Earth's biosphere (Knoll and Nowak 2017), life on Mars, if present, may have been limited to unicellular forms and simple metabolisms such as chemolithotrophy (Onstott et al. 2019; Westall et al. 2025). Iron‐based metabolisms have been considered a potential energy source for life on early Mars owing to the abundance of iron and varying redox conditions on the ancient surface (Hurowitz et al. 2017; Weber et al. 2006). Most cell‐sized morphological biosignatures would be too small to be resolved by current in situ imaging capabilities of instruments onboard Mars exploratory craft (McMahon et al. 2018), but the prospect of sample return in the future could facilitate microfossil identification in extraterrestrial material (Clodoré et al. 2024; Haltigin et al. 2022; Williford et al. 2018). Larger microbial extracellular structures on the order of tens of micrometres in size may be possible to detect using in situ instrumentation (McMahon et al. 2018; Onstott et al. 2019). Notably, iron‐mineralised chemical gardens resemble some of these larger extracellular structures, especially those produced by iron‐metabolising bacteria (McMahon 2019). Therefore, iron‐mineralised chemical garden production may be an important abiotic mechanism to be considered for the evaluation of any microstructures in Martian samples posited as biogenic in the future.

For interpretation of the microfossil record, understanding the effects of diagenesis is critical as post‐depositional alteration can obscure diagnostic morphologies and stimulate the formation of abiotic biomorphs (Criouet et al. 2021; Javaux 2019). Artificial maturation can be used to elucidate these effects, so that they can be accounted for when evaluating the origins of microstructures that resemble biology in the rock record. For example, organic biomorphs have been shown to be better preserved in siliceous rocks than microorganisms, which underscores their potential to act as pseudofossils (Nims et al. 2021). The preservation potential of iron‐mineralised microfossils has been shown to vary with the original morphology of the structures (Hirsch et al. 2025), and a diverse range of morphologies has been previously described (e.g., Parenteau and Cady 2010). Artificial maturation of ferrihydrite twisted stalks produced by iron‐oxidising bacteria demonstrated the resistance of the structures to degradation through maturation (Picard et al. 2015). In contrast, ferrihydrite‐rich extracellular sheaths of iron‐oxidising bacteria were shown to be more susceptible to degradation (Hirsch et al. 2025). This suggests that the preservation potential of such structures may depend on the presence of a suitable mineral medium (such as silica/chert) to minimise morphological disruption. To date, the preservation potential of iron‐mineralised chemical gardens has not been investigated, which increases uncertainty as to whether they offer a feasible explanation for the origin of morphologically similar structures in matured rocks.

Despite the challenges involved in simulating geological processes on practical timescales in the laboratory, various methods exist to reproduce the chemical and mechanical effects of diagenesis. Raising the temperature and/or pressure of a sample for short periods of time can be considered kinetically analogous to geological processing in less energetic conditions over longer time periods (Lewan et al. 1979). Hydrous pyrolysis is a laboratory technique for artificial maturation based on this principle, whereby a sample is heated in a sealed vessel under aqueous, anoxic conditions. Though commonly used to investigate chemical changes, typically of organic matter (Lewan et al. 1979; Tan and Sephton 2021), hydrous pyrolysis has been employed to understand the preservation of morphological biosignatures and their modification by diagenetically induced mineralogical transformations (Hirsch et al. 2025).

In this study, we aimed to elucidate the preservation potential of iron‐mineralised chemical gardens and identify chemical changes induced during their diagenesis, in order to aid evaluation of the origin of fossilised iron‐mineralised filamentous microstructures. If these structures turn out to decompose readily upon heating, their potential to confound the recognition of true fossils is correspondingly minimised. On the other hand, if they are less liable to decomposition than iron‐oxidising bacterial sheaths, their importance as pseudofossils is correspondingly increased. Thus, our findings could be especially critical for interpretation of some of the oldest purported microfossils, and potentially morphological evidence for extraterrestrial life.

## 2 Methods

### 2.1 Growth of Chemical Gardens

Chemical gardens were produced according to the methodology outlined by McMahon (2019). Polycrystalline granules of iron(II) sulfate heptahydrate (98% FeSO_4_ · 7H_2_O; Alfa Aesar, Heysham, UK) were sieved to obtain the grain‐size fraction below 63 μm. These grains were scattered in Petri dishes to which aqueous solutions of 100 g L^−1^ sodium silicate (prepared from powder containing 53% SiO_2_, 26% Na_2_O, Scientific Laboratory Supplies, Nottingham, UK; pH 12.1) were then added, submerging the grains. The experiment was not stirred or agitated. After 24 h, the solution was replaced with distilled water by pipetting (three times) and then allowed to dry at room temperature and pressure (drying was complete within 3 days). These conditions were chosen as previously shown to produce life‐like chemical gardens similar in size and shape to the sheaths of iron‐oxidising bacteria and to various putative microfossils (McMahon 2019; Podbielski et al. 2025).

### 2.2 Artificial Maturation of Chemical Gardens

Hydrous pyrolysis was used to artificially mature the chemical garden samples. Roughly 5 mg of chemical garden material was loaded into seven borosilicate glass tubes with 0.4 mL of ultrasonically degassed, deionised water. The glass tubes were then frozen using liquid nitrogen, and flame sealed under vacuum to eliminate air (and molecular oxygen) from the headspace of the tubes. Each glass tube was then placed inside a 75 mL 4740 stainless steel high‐pressure reactor (Parr Instrument Company) with 30 mL of deionised water added into the bomb to ensure equal pressure along the walls of the glass tube. The reactor was then heated to a range of temperatures up to 350°C for 72 h. These temperatures are listed in Table 1. The associated pressure was estimated as the vapor pressure of water at the given reactor temperature, assuming the volume of any gases produced from the chemical garden material during pyrolysis to be negligible.

**TABLE 1:** Hydrous pyrolysis temperatures used to artificially mature the chemical garden samples with associated estimated pressures.

Hydrous pyrolysis temperature (°C)	Estimated pressure (MPa)
50	—
100	0.1
150	0.5
200	1.6
250	4.0
300	8.7
350	16.6

### 2.3 Scanning Electron Microscopy and Energy Dispersive X‐Ray Spectroscopy

Samples were placed onto stubs covered with carbon tape and loaded onto the sample stage for observation using a Hitachi TM4000 scanning electron microscope (SEM). Backscattered electron (BSE) images were acquired at 15 kV and an average working distance of 7 mm. Elemental analysis was performed using an Oxford Instruments energy dispersive X‐ray spectroscopy (EDS) system integrated with the SEM. EDS data was analysed with Oxford Instruments AZtec software.

### 2.4 Quantification of Changes in Chemical Garden Morphology

For each sample, up to 50 SEM photomicrographs were taken at x500 magnification. If the length of a filament exceeded the field of view then lower magnifications were employed. The photomicrographs were imported into ImageJ computer software to measure the filament lengths and widths, with the scale bar used to calibrate the measurements. Only structures that clearly exhibited filamentous morphologies were measured. If the structures were branched, the length of the longest branch was measured. If the width of the filament varied along its length, the maximum width was measured. The lengths and widths of 300 filaments were measured for each sample.

An independent, two‐tailed Student's *t*‐test was performed for the length and width data between each artificially matured sample and the unheated sample. A *p*‐value of < 0.05 indicated statistically significant variation from the unheated sample.

### 2.5 X‐Ray Diffraction

Powdered samples were mounted with glue between two Kapton foils and fixed into inserts with a 3 mm circular mask. The inserts were placed on the diffractometer sample stage which was rotated during the measurements. X‐ray diffraction measurements were conducted using a STOE STADI MP powder diffractometer equipped with an incident‐beam monochromator and a Mythen 1 K detector (DECTRIS). A molybdenum X‐ray source was used at 50 kV and 40 mA. Diffraction data were collected in transmission geometry over an angular range of 2 to 39.5°2*θ*, with a step size of 0.015° and data collection times between 2300 and 5000 s per step. Phase identification was performed using the Highscore software (Malvern Panalytical) with reference patterns from the COD and PDF‐5 databases.

## 3 Results

### 3.1 Microscopic Analysis of Artificially Matured Chemical Garden Material

**FIGURE 1:**
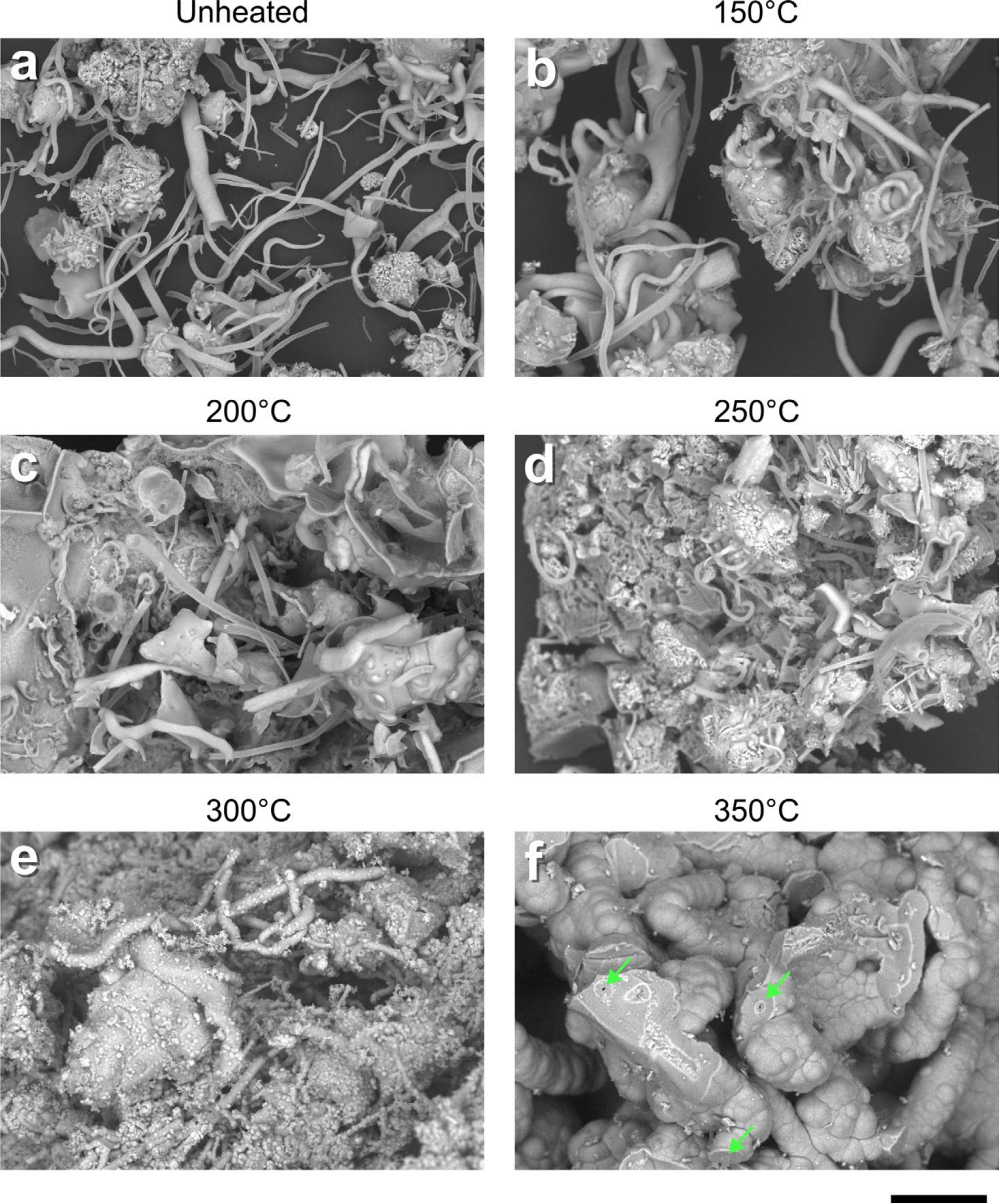
SEM photomicrographs of unheated (a) and artificially matured (b–f) iron‐mineralised chemical garden material with the hydrous pyrolysis temperature given above each sample. Filamentous material is clearly discernible in (a–e). In (f), green arrows point to bright circles identified as possible cross sections of remnant filaments. Scale bar = 50 μm.

In the unheated sample, the chemical garden material exhibited a range of morphologies, similar to those observed in previous studies (Figures 1a and S1) (McMahon 2019; Podbielski et al. 2025). The sample was dominated by tubular (hollow) filamentous material alongside remains of the globular envelopes initially formed around the seed grains. While some filaments varied in diameter along their length and tapered ends were common, many had consistent external diameters and circular cross sections. Additionally, some of the filaments displayed branching and anastomosis (Figure S1).

With increased temperature of artificial maturation the filaments became more clumped, and often formed large clusters in combination with the seed grain envelope remnants and rather shapeless material (Figure 1b–f). In some cases, this meant that the filaments were harder to discern, but generally many of the structures could still be clearly recognised in all samples treated at temperatures ≤ 300°C. In the sample treated at 300°C (Figure 1e), roughly spheroidal microstructures with diameters of 0.5–2.5 μm (microspheres) were observed throughout the sample, often coating the filaments. The sample treated at 350°C was dominated by bulbous/botryoidal material, sometimes forming larger spheres. The traces of some filaments were identified in this sample, with bright circles in the BSE images likely to be a cross section of the iron‐rich filaments (Figure 1f). However, entire filaments could not be clearly discerned in this sample because they were embedded in the bulbous/botryoidal material (Figure 1f).

**FIGURE 2:**
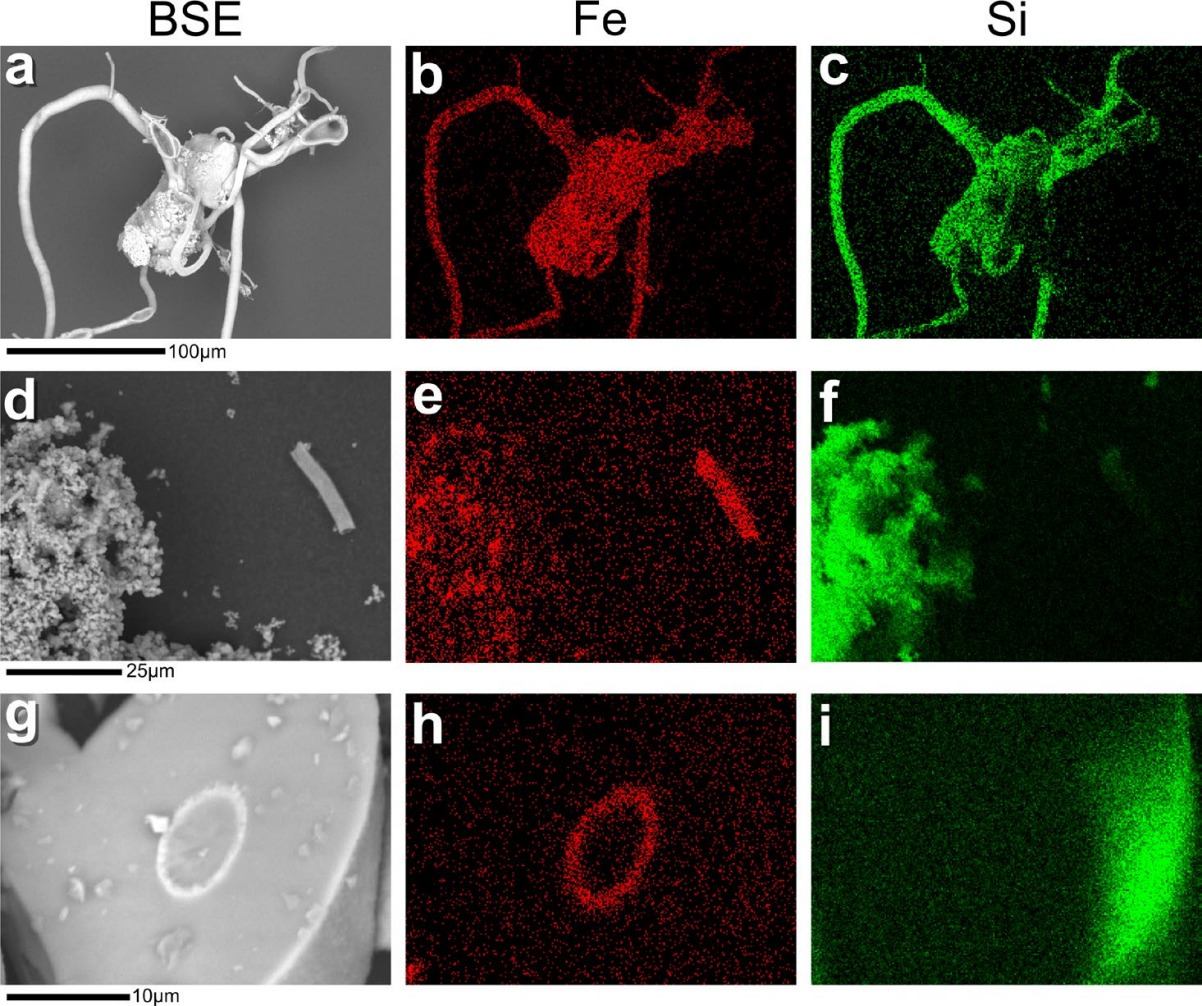
SEM photomicrographs and EDS elemental maps of unheated and artificially matured iron‐mineralised chemical garden material. The heading above each column indicates the type of images below: BSE = backscattered electron photomicrograph, Fe = iron EDS elemental map, Si = silicon EDS elemental map. (a–c) Filaments and seed grain remnant from the unheated material. (d–f) Isolated filament and microspheres from the sample treated at 300°C. (g–i) Potential filament cross section in the sample treated at 350°C.

Energy dispersive X‐ray spectroscopy (EDS) maps of the unheated chemical garden material showed the elemental composition of the filaments and seed grain remnants to be dominated by iron and silicon (Figure 2a–c). In the sample treated at 300°C, isolated filaments were also composed of iron but appeared relatively silicon‐poor (Figure 2d–f). This was supported by EDS point spectra that revealed the elemental composition of the filaments and seed grains to be predominantly iron, oxygen and silicon, whereas the microspheres were composed only of oxygen and silicon (Figure S2). EDS elemental maps indicated that the bright circles noted in the SEM photomicrographs of the sample treated at 350°C were enriched in iron, supporting their identification as filament remains (Figure 2g–i).

### 3.2 Changes in Morphological Parameters With Increasing Maturity

**FIGURE 3:**
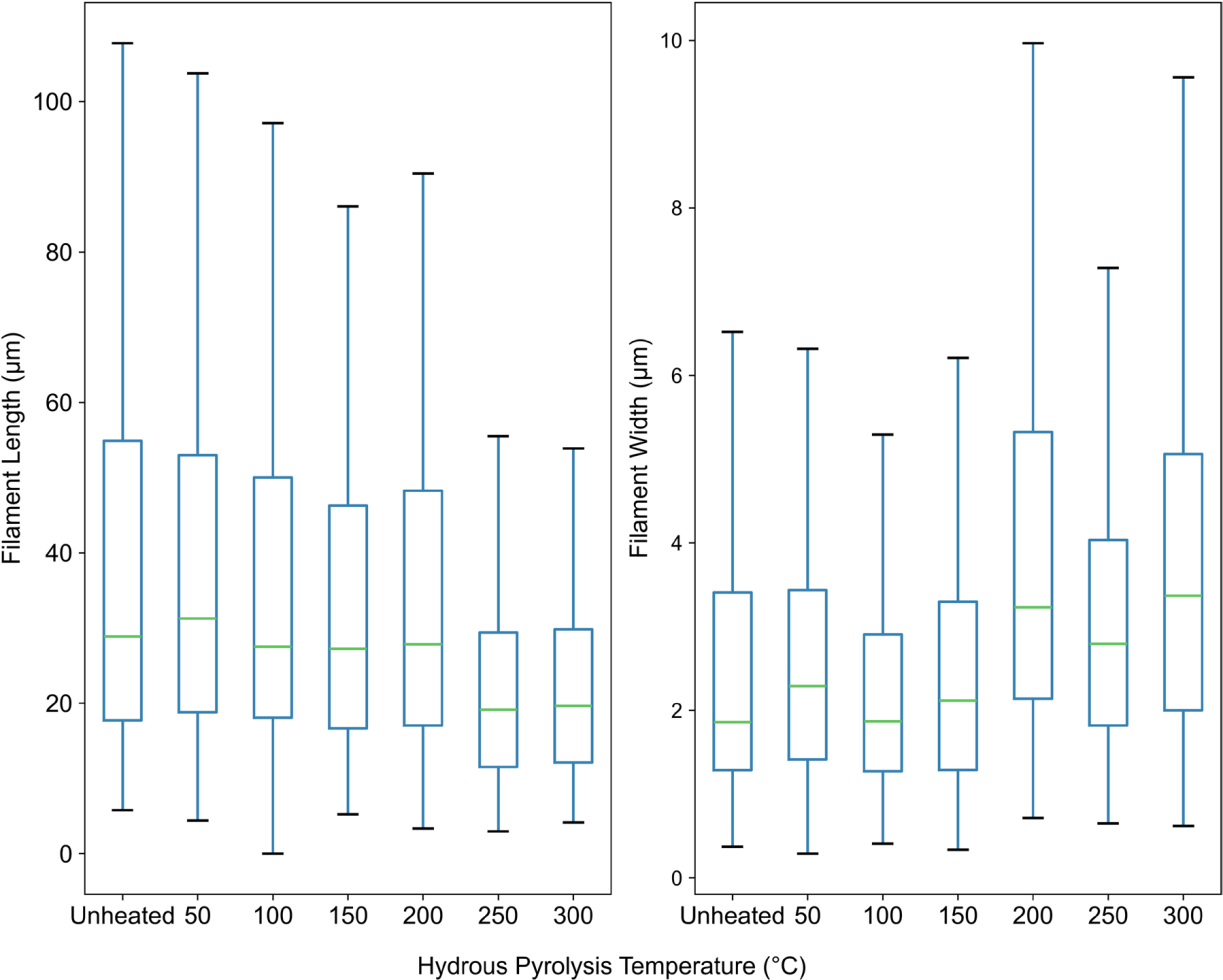
Boxplots representing the distribution of filament lengths and widths (*n* = 300) in the iron mineralised chemical garden samples treated at increasing hydrous pyrolysis (artificial maturation) temperatures. Whiskers are 1.5 times the interquartile range and outliers beyond this are omitted.

The distribution of the lengths and widths of the filaments in each sample is shown in Figure 3. The mean, maximum, minimum, and standard deviation of the length and width distributions are shown in Tables 2 and 3 respectively, alongside the results of the Student's *t*‐tests.

**TABLE 2:** Changes in chemical garden filament lengths (*n* = 300) measured in each sample with increasing temperature of hydrous pyrolysis (artificial maturation).

Hydrous pyrolysis temperature (°C)	Unheated	50	100	150	200	250	300
Mean (μm)	46.2	42.39	41.77	39.23	41.6	24.94	28.79
Standard deviation (μm)	50.49	37.59	44.98	42.03	41.43	21.18	32.75
Maximum (μm)	390.77	258.92	399.62	494.64	309.27	201.59	240.06
Minimum (μm)	5.78	4.40	4.56	5.21	3.36	2.96	4.16
*p* (unpaired *t*‐test against *F*‐unheated)	—	0.30	0.26	0.07	0.22	< 0.00001	< 0.00001

*Note:* No filaments could be discerned clearly enough for measurement in the sample treated at 350°C.

**TABLE 3:** Changes in chemical garden filament widths (*n* = 300) measured in each sample with increasing temperature of hydrous pyrolysis (artificial maturation).

Hydrous pyrolysis temperature (°C)	Unheated	50	100	150	200	250	300
Mean (μm)	2.63	2.66	2.45	2.55	4.06	3.26	3.83
Standard deviation (μm)	0.37	0.29	0.41	0.34	0.71	0.65	0.62
Maximum (μm)	10.48	9.53	10.71	10.4	15.69	17.25	11.88
Minimum (μm)	1.95	1.63	1.89	1.74	2.74	2.11	2.34
*p* (unpaired *t*‐test against *F*‐unheated)	—	0.81	0.28	0.64	< 0.00001	< 0.00001	< 0.00001

*Note:* No filaments could be discerned clearly enough for measurement in the sample treated at 350°C.

**TABLE 4:** Summary of mineralogical changes of chemical garden material with increasing hydrous pyrolysis (artificial maturation) temperature based on XRD results (Figure 4).

Hydrous pyrolysis temperature (°C)	Iron phase	Silica phase
Unheated	Ferrihydrite	Not observed
50–250	Ferrihydrite	Not observed
300	Possible haematite	Cristoballite and Tridymite
350	Not observed	Cristoballite and Tridymite

*Note:* Silica phases in low temperature samples are likely not observed because they are amorphous. Iron phases in the high temperature samples are likely poorly observed due to peak overlaps with the highly crystalline silica phases.

Student's *t*‐tests did not detect statistically significant (*p* < 0.05) changes in the distribution of filament lengths between the unheated sample and samples treated at temperatures ≤ 200°C (*n* = 300). For samples treated at temperatures ≥ 250°C, the lengths of the filaments significantly decreased relative to the unheated sample (*n* = 300).

For the width measurements, Student's *t*‐tests did not detect a significant change between the unheated sample and samples treated at temperatures ≤ 150°C (*n* = 300). For samples treated at temperatures ≥ 200°C, the widths of the filaments significantly increased relative to the unheated sample (*n* = 300).

### 3.3 Mineralogical Changes

**FIGURE 4:**
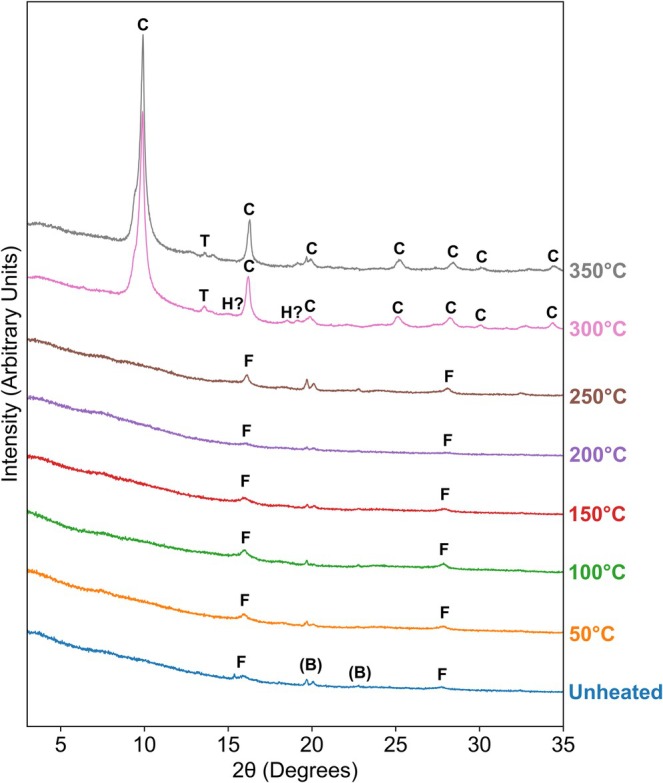
XRD patterns of unheated and artificially matured iron‐mineralised chemical garden samples. Coloured numbers on the right‐hand side indicate the hydrous pyrolysis temperature. Peaks are labelled with the minerals they were associated with based on the reference database: F = ferrihydrite, C = cristobalite, T = tridymite, H = haematite. (B) indicates peaks identified in the blank sample originating from the equipment. Mineralogical changes are summarised in Table 4. Individual XRD patterns for representative samples are shown in Figures S3 and S4.

To elucidate mineralogical changes associated with artificial maturation experiments, X‐ray diffraction (XRD) patterns were obtained from the samples and are displayed in Figure 4. An XRD pattern of a blank sample with the foil and glue used to prepare the samples was also taken (Figure S3a) and the peaks identified in this pattern were disregarded from the interpretation of the sample XRD patterns. The XRD pattern of the unheated sample exhibited two broad peaks associated with poorly crystalline two‐line ferrihydrite. This pattern is shown isolated in Figure S3b to aid visual recognition of these broad peaks. For samples treated at temperatures ≤ 250°C, the XRD pattern did not vary significantly from the unheated sample with the same two broad ferrihydrite peaks identified in each pattern. For the samples treated at 300°C and 350°C, high intensity peaks were associated with cristobalite and tridymite. In these samples, no peaks could be confidently associated with an iron‐bearing phase. In the sample treated at 300°C, two low intensity peaks were tentatively identified as haematite (Figure S4).

## 4 Discussion

### 4.1 Preservation Potential of Iron‐Mineralised Chemical Garden Filaments

Our experiments demonstrated the preservation potential of iron‐mineralised chemical garden filaments through thermal maturation under aqueous, anoxic conditions, in the absence of an entombing medium. Generally, the filaments were resistant to moderate diagenesis and only became significantly obscured in samples treated at 300°C–350°C.

The significant decreases in the distributions of the lengths of the filaments following treatment at high pyrolysis temperatures are likely a result of breakages caused by thermal stress induced by the artificial maturation process, and are therefore a useful quantitative representation of the extent of degradation. The significant increase in the widths of the filaments at higher hydrous pyrolysis temperatures is perhaps unexpected when compared to the decrease in lengths found for these samples, and the general expectation for maturation to cause degradation of microstructures. This may be a result of silica coating the filaments, or preferential destruction of thinner filaments at higher temperatures of pyrolysis.

Previously, Hirsch et al. (2025) used hydrous pyrolysis to investigate the preservation of bacterial iron‐mineralised sheaths. Because the method of artificial maturation was consistent with this study, the preservation potential of the abiotic chemical garden structures can be directly compared to that of morphologically and chemically similar biological material. The biological sheaths were produced by *Leptothrix ochracea* , a chemolithotrophic iron‐oxidising bacteria found in circumneutral iron‐rich environments. The structural and compositional similarity of *L. ochracea* sheaths and iron‐mineralised chemical gardens has recently been reviewed, with important consequences for the interpretation of the microfossil record due to the potential for false positive conclusions of biogenicity (Podbielski et al. 2025). We consider this comparison critical to interpreting the results of this study and will refer to the results of bacterial sheath preservation experiments by Hirsch et al. (2025) in the following discussion.

In comparison to the bacterial sheath structures, iron‐mineralised chemical garden filaments consistently exhibited greater preservation potential under the conditions tested. Firstly, bacterial sheaths could not be observed following artificial maturation at 280°C, whereas chemical garden filaments could be clearly distinguished in the sample treated at 300°C. Secondly, a significant decrease in the distribution of the artificially matured biological sheath lengths versus the unheated sample was observed at 150°C, while filament lengths in the chemical garden sample showed a significant decrease at 250°C.

### 4.2 Changes in Mineralogy

The unheated chemical garden filaments were shown by XRD analysis to be dominated by poorly crystalline two‐line ferrihydrite, in accordance with previous studies (McMahon 2019; Podbielski et al. 2025). Ferrihydrite is an iron oxyhydroxide mineral, metastable at terrestrial surface conditions but stable and abundant on the Martian surface (Cudennec and Lecerf 2006; Dehouck et al. 2017; Valantinas et al. 2025). Upon diagenesis or artificial heating, ferrihydrite transforms into more crystalline iron minerals (Johnston and Lewis 1983; Posth et al. 2013; Ristić et al. 2007). Under oxidising conditions, the resulting phase is typically haematite or goethite, whereas in more reducing conditions maghemite or magnetite form (Campbell et al. 1997).

In our experiments, ferrihydrite was stable at hydrous pyrolysis temperatures of at least 250°C and no clear signal for further ferruginous minerals was identified in the XRD patterns. This is in contrast with the results of previous hydrous pyrolysis experiments that observed the transformation of synthetic and biogenic ferrihydrite to magnetite at 100°C and 150°C respectively (Tan and Sephton 2021).

For samples treated at ≥ 300°C, high intensity, well‐defined peaks associated with cristobalite and tridymite phases were identified in the XRD patterns. Although cristobalite and tridymite are considered high‐temperature polymorphs of silica, their presence in these samples is expected because they typically form at lower temperatures as a metastable intermediate in the hydrothermal transformation of amorphous silica to quartz (Bettermann and Liebau 1975). It is likely that amorphous silica was present in the unheated sample because EDS elemental maps indicated the presence of silicon (Figure 2a–c) but no peaks associated with crystalline silica phases were observed in the XRD patterns (Figure 4). In contrast to the unheated samples, isolated filaments in the sample treated at 300°C were relatively depleted in silicon (Figure 2f). This suggests that the artificial maturation caused amorphous silica originally associated with filaments to be redistributed to form the microsphere structures.

The presence of silica(te) species has been shown to inhibit the transformation of ferrihydrite by adsorbing onto and linking particles of the mineral, hindering the nucleation of crystalline phases (Cornell et al. 1987; Jones et al. 2009). The presence of silica(te) is inherent to the formation of the iron‐mineralised chemical garden filaments as they incorporate silicate ions onto their exterior surfaces from the sodium silicate solution in which they are produced (Kotopoulou et al. 2021; McMahon 2019). This is also evident from the presence of silicon throughout the filament structures in the EDS elemental map of the unheated sample (Figure 2c). Therefore, the presence of this silica(te) in close proximity with the ferrihydrite likely inhibits this diagenetic transformation. Aside from two minor peaks tentatively associated with haematite (Figures 4 and S4), for the samples treated at ≥ 300°C, no XRD peaks were observed for ferrihydrite, or for more crystalline iron oxide phases that it may be transformed to. Given that an iron‐bearing phase must still be present in the samples, and elemental maps indicate the presence of iron‐rich filament remains (Figure 2g–i), it is likely that the XRD peaks from the ferruginous mineral(s) were masked by overlapping high‐intensity peaks for the crystalline silica phases.

In the sample treated at 300°C, the formation of microsphere structures in the SEM photomicrographs occurred alongside the detection of silica polymorph peaks in the XRD. Combined with the indication of a lack of iron in the microspheres in EDS point spectra (Figure S2), it can be assumed that the microsphere structures are formed of cristobalite and tridymite. As the iron bearing phase was likely obscured by the crystalline silica signal in XRD data for these samples, the mineralogy of the filaments in these samples cannot be confidently established. Therefore it is unknown whether silica inhibited the transformation of ferrihydrite at these temperatures or if a phase transformation was induced but could not be observed in the XRD data.

As the extent of silica polymorph crystallisation was so broad in the sample treated at 350°C (Figure 1f), it is unclear if the filaments have been degraded as well as obscured. Some traces of highly silicified filaments and their cross sections were visible (Figures 1f and 2g–i), but overall filamentous material was difficult to discern and could not be identified with sufficient confidence for dimensions to be measured. This highlights the potentially complex effects of silica on the preservation of the filaments. Because diagenetic recrystallisation has been linked with degradation of similar microstructures (Hirsch et al. 2025; Lima‐Zaloumis et al. 2024), the presence of silica may increase the preservation potential of the filaments by inhibiting the transformation of ferrihydrite. Furthermore, in terrestrial rocks, silica has facilitated exceptional microfossil preservation (Chi Fru et al. 2013; Javaux 2019). Conversely, the formation of the crystalline silica polymorph material, as a result of the presence of silica, obscures the observation of the filament structures by SEM. Other analyses such as transmission‐electron microscopy or petrographic thin section microscopy may have the potential to overcome this obscuration effect by imaging sample cross sections rather than the bulk material.

The diagenetic transformation of ferrihydrite to magnetite was proposed as a potential cause of degradation of the bacterial sheaths (Hirsch et al. 2025). In comparison, a significant decrease in the lengths of the filaments from the unheated sample was detected by the Student's *t*‐test in the chemical garden sample treated at 250°C, despite the mineralogy of the sample being unchanged from the original ferrihydrite. This indicates that significant degradation occurred without an induced phase transformation. Further study of the influence of silica on the preservation of other ferrihydrite materials, such as the sheaths of *L. ochracea* , would help to constrain the degradational effects of diagenetic recrystallisation.

### 4.3 Implications for the Interpretation of the Terrestrial Microfossil Record

It should be emphasised that the procedure used to generate chemical gardens in our experiments is not geochemically naturalistic. It is still an open question whether any iron‐mineral filaments encountered in the environment are true chemical gardens. However, García‐Ruiz et al. (2017) have shown that naturally occurring silica‐rich springwaters can produce chemical gardens similar to those produced here. McMahon et al. (2021) likewise showed that the mineral products of sulfide oxidation can serve as suitable ‘seeds’. Silva‐Bedoya et al. (2021) showed that the ‘rusticles’ found on corroding ships are very rich in silica (as are the structures used here) and also likely to represent chemical gardens.

The potential for chemical garden filaments to survive substantial diagenetic processing as demonstrated in this study implies a need for caution in the interpretation of the microfossil record. Compared to some of the most morphologically similar biological material (Hirsch et al. 2025), chemical garden filaments are more resistant to degradation through maturation. Therefore, in highly matured rocks, the chance of false‐positive claims of biogenicity based solely on iron‐mineralised filamentous morphology is enhanced, as diagenetic processes will bias preservation of the abiotic material over the biotic. These findings may be especially significant for the interpretation of some of the most ancient microfossils, many of which consist of iron‐mineralised filaments, often hosted in siliceous matrices (Dodd et al. 2017; Papineau et al. 2022; Rasmussen 2000; Shapiro and Konhauser 2015). Compared to their younger counterparts, these ancient microfossils are more likely to be found in highly matured rocks and are less likely to be associated with other evidence for biogenicity such as organic matter. Furthermore, many of the most primitive microfossils are found in hydrothermal vent deposits (e.g., Dodd et al. 2017), environments that are plausibly conducive to the formation of chemical garden material—although this has not been demonstrated directly (Johannessen et al. 2020; McMahon 2019).

Careful comparison of microscopic images of bacterial sheaths and chemical garden filaments has enabled the identification of features that may disambiguate their similar morphologies (Podbielski et al. 2025). Compared to sheaths of *Leptothrix* bacteria, chemical garden filaments are typically more varied in diameter. Our results suggest that before artificial maturation, chemical garden filaments are also significantly greater in length than their biotic counterparts. However, using size to distinguish biotic sheaths from abiotic chemical garden filaments may be unreliable as chemical garden filaments have been observed forming at a range of sizes depending on the seed grain size and the nature of local fluid flow (McMahon 2019; Podbielski et al. 2025). Moreover, as the chemical garden filaments were artificially matured, the mean and standard deviation of their lengths decreased, further confounding this size distinction. On the other hand, because the widths of the chemical garden filaments were increased following high temperature pyrolysis, maturation processes may facilitate their distinction from *Leptothrix* sheaths based on their diameter. A further potentially distinguishing feature is the contrasting surface textures: bacterial sheaths have rough exterior surfaces and smooth interiors, whereas chemical garden filaments display the inverse (Podbielski et al. 2025). It is difficult to tell how well these distinctive textures are preserved through simulated diagenesis with the resolution of the microscopy used herein, but clearly the silicification observed in the samples treated at high temperatures serves to inhibit observation of the surface textures by SEM.

### 4.4 Consequences for the Interpretation of Extraterrestrial Biosignatures

The risk of making false‐positive claims of biogenicity is especially acute in the field of astrobiology. Already, morphology has been used as evidence for extraterrestrial life (McKay et al. 1996) and subsequently dismissed when abiotic mechanisms provided more plausible explanations for the origin of the biomorphic microstructures (Golden et al. 2001). Plausible formation of iron‐mineralised chemical gardens has been proposed in extraterrestrial settings including the rock‐water interface in the interiors of icy moons (Barge et al. 2015; Barge and White 2017) and the aqueous surface of early Mars (Sainz‐Díaz et al. 2021).

The consideration of chemical garden formation on Mars is especially pertinent due to performance of in situ analyses by present and future missions in the search for traces of life on the planet (e.g., Farley et al. 2020; Vago et al. 2017). The sheaths of *Leptothrix* bacteria have been highlighted for their importance as a potential analogue for Martian morphological biosignatures (Hirsch et al. 2025). As the morphologies of chemical garden filaments mimic these biotic sheath structures, consideration of their potential for formation and preservation is paramount. The formation of aluminium‐based chemical gardens in simulated aqueous (modern) Martian P–T conditions has been demonstrated (Sainz‐Díaz et al. 2021). More generally, chemical garden formation in early Martian environments has been hypothesised due to the widespread potential for the interaction between alkaline fluids and metal salts (McMahon 2019; McMahon et al. 2021). Our demonstration of the high preservation potential of the precipitated tubes and filaments further highlights the potential for chemical gardens to serve as an abiotic hypothesis when evaluating potentially biogenic filamentous microstructures that may be found in Martian samples in future.

As discussed, larger filamentous microstructures may be detectable with current in situ imaging capabilities on Mars (Onstott et al. 2019). For instance, the Mars Hand Lens Instrument (MAHLI) onboard the Mars Curiosity rover has a pixel size of 13.9 μm which is capable of discerning mat‐like textures formed by microbial mineral filaments (Williams et al. 2015). However, the resolution of these instruments is certainly insufficient to reliably distinguish biotic and abiotic iron‐mineralised morphologies. Therefore, in situ detection of ferruginous filament structures would warrant further analyses and sampling, potentially for return to Earth (Bosak et al. 2021; Clodoré et al. 2024). With the higher resolving power of terrestrial microscopic analyses, morphology could then constitute a vital line of evidence towards establishing the presence of fossil life in returned Martian samples. The preservation of organic biosignatures in iron‐rich Martian environments has been shown to be highly variable depending on regional chemical conditions (Tan et al. 2018; Tan and Sephton 2021). For instance, in circumneutral Martian environments dominated by ferrihydrite, kinetic modelling suggested that no solvent extractable organic matter would remain in biological deposits formed during the period of aqueous surface conditions (Tan and Sephton 2021). Consequently, the absence of chemical biosignatures in iron‐rich Martian samples could heighten the need to confidently evaluate biogenicity based on morphology alone.

As the original morphology and mineralogy of microstructures plays a critical role in their preservation potential, the observed resistance of iron‐mineralised chemical garden filaments to degradation through maturation should not be generalised to the broad chemical and structural diversity of all chemical garden materials. Further artificial maturation experiments of other chemical garden mineralogies and morphologies would facilitate a more generalised understanding of their preservation potential. Likewise, the sheaths produced by *L. ochracea* were selected for comparison because of their similarity to chemical garden material, but should not be considered representative of all biological microstructures. Furthermore, extraterrestrial morphological biosignatures could differ entirely beyond any known from the fossil record on Earth. Despite these limitations, the chemical gardens in this study and the comparison with the sheaths of iron‐oxidising bacteria remain a demonstrative example of the relative preservation potential of abiotic and biotic ferruginous filaments which may be critical for the interpretation of similar extraterrestrial microstructures if they are discovered in the future.

Finally, iron‐mineralised chemical gardens have been considered for their relevance to prebiotic chemistry (Barge et al. 2015; Kotopoulou et al. 2021). The high porosity and surface area of the structures provide them with the potential to catalyse prebiotic organic reactions (Collins et al. 1999), and nanoscale bilayer membrane features within the material elicit the potential for primitive cellular organisation (Kotopoulou et al. 2021). Together, these catalytic and compartmentalisation properties suggest a role for chemical gardens as precursors to the first protocell (Monnard and Walde 2015; Ruiz‐Mirazo et al. 2014), especially given their potential formation in hydrothermal vent systems thought to be abundant on the early Earth and Mars (Johannessen et al. 2020). Hence, any future observations of preserved chemical garden structures must first be carefully distinguished from biotic materials, but could subsequently be considered for their potential role in abiogenesis.

## 5 Conclusion

In summary, we have investigated the morphological and mineralogical changes induced during the artificial maturation of iron‐mineralised chemical garden material. Degradation caused by maturation of the chemical garden filaments was relatively limited, especially in comparison to similar biological materials. Also, in comparison to biological filaments, the chemical garden filaments retained their original ferrihydrite mineralogy under higher intensity maturation. As the preservation potential of the abiotic filaments has now been demonstrated, these results motivate careful scrutiny of fossilised iron‐mineralised filaments, which must be distinguished from the abiotic alternative in order for their biogenicity to be confidently established. Ideally, further lines of evidence such as chemical or isotopic biosignatures should be investigated to confirm a biogenic origin of such materials. If these data are lacking, detailed morphological analyses and consideration of geological context may have the potential to disambiguate biotic filamentous assemblages. These considerations could be useful for the evaluation of candidate microfossils found in terrestrial rocks, on planetary surfaces by in situ astrobiological observations, or in extraterrestrial samples brought to Earth in the future.

## Funding

This work was supported by the UK Space Agency (Grants ST/V002732/1, ST/V006134/1, and ST/Y006194/1).

## Conflicts of Interest

The authors declare no conflicts of interest.

## Supporting information


**Figure S1:** SEM photomicrographs of unheated iron‐mineralised chemical garden samples displaying broad morphological diversity. Anastomosis (a), branching (b) and helical structures (c) are visible and labelled with green arrows. Scale bars = 50 μm.
**Figure S2:** (a) Photomicrograph of iron‐mineralised chemical garden material artificially matured at 300°C with spectra areas labelled. Spectrum 1 (orange) was targeted at an isolated group of microspheres. Spectrum 2 (blue) was targeted at the filamentous material (b) EDS spectra acquired from ellipses labelled in photomicrograph with peaks labelled with associated elements. Scale bar = 50 μm.
**Figure S3:** (a) XRD pattern of the background signal from the foil and glue used for sample preparation. (b) XRD pattern of unheated iron‐mineralised chemical garden sample showing the same background peaks alongside two peaks matching those of ferrihydrite in the reference database (labelled F).
**Figure S4:** XRD pattern of the sample artificially matured at 300°C. Reference peaks for tridymite, haematite and cristobalite used for peak assignment are shown below.

## Data Availability

The data that support the findings of this study are available from the corresponding author upon reasonable request.
