# Supplementary material for: Tricks of Nature From the Ancient Earth and Early Mars: Chemical Gardens Generate Biomorphs With High Preservation Potential
Source: Geobiology. 2026 Apr 21;24:e70045. doi: 10.1111/gbi.70045 (PMC13100049; doi:10.1111/gbi.70045)
Supplement: Supplementary file 1 — Figure S1: SEM photomicrographs of unheated iron‐mineralised chemical garden samples displaying broad morphological diversity. Anastomosis (a), branching (b) and helical structures (c) are visible and labelled with green arrows. Scale bars = 50 μm. Figure S2: (a) Photomicrograph of iron‐mineralised chemical garden material artificially matured at 300°C with spectra areas labelled. Spectrum 1 (orange) was targeted at an isolated group of microspheres. Spectrum 2 (blue) was targeted at the filamentous material (b) EDS spectra acquired from ellipses labelled in photomicrograph with peaks labelled with associated elements. Scale bar = 50 μm. Figure S3: (a) XRD pattern of the background signal from the foil and glue used for sample preparation. (b) XRD pattern of unheated iron‐mineralised chemical garden sample showing the same background peaks alongside two peaks matching those of ferrihydrite in the reference database (labelled F). Figure S4: XRD pattern of the sample artificially matured at 300°C. Reference peaks for tridymite, haematite and cristobalite used for peak assignment are shown below. [file GBI-24-e70045-s001.docx]

**Supplementary Information**


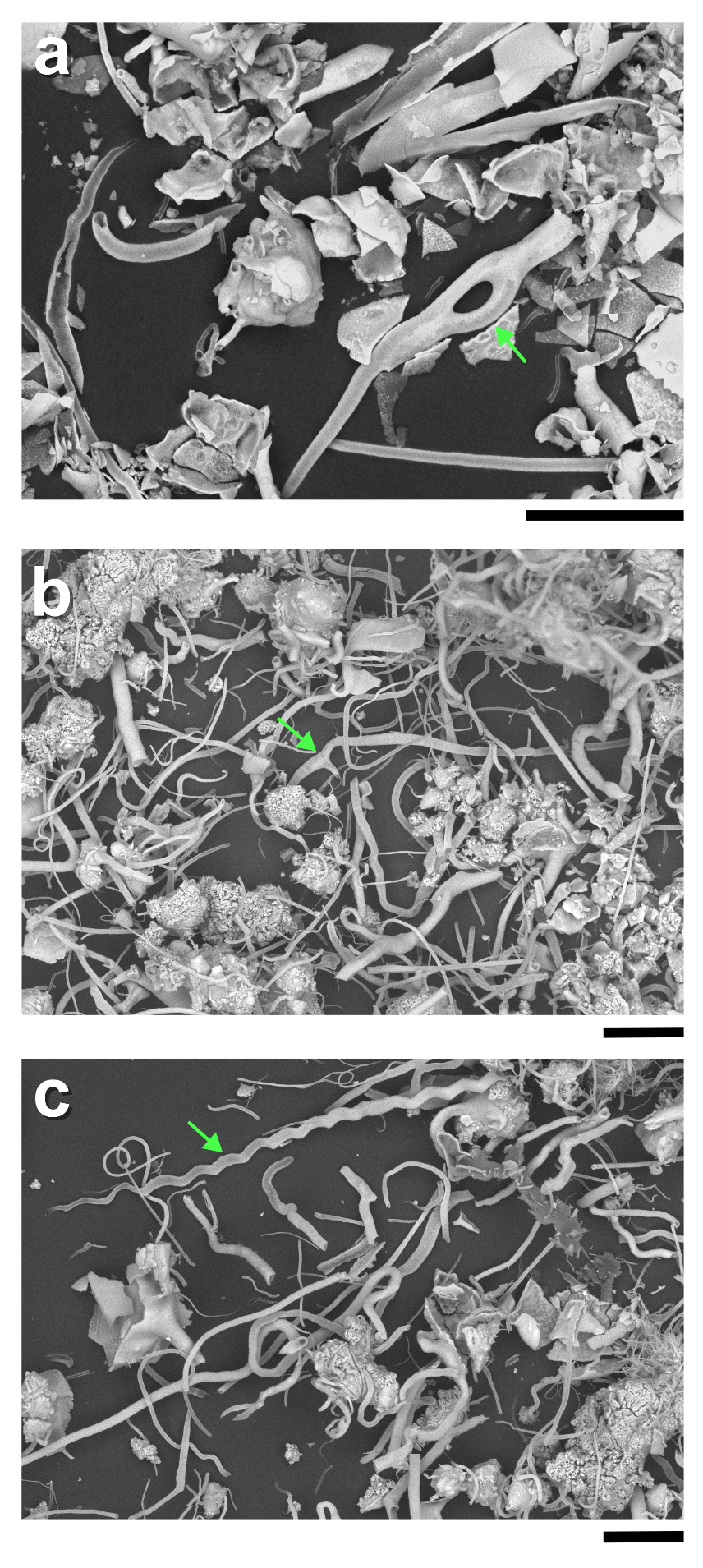


**Supplementary Figure 1:** SEM photomicrographs of unheated iron-mineralised chemical garden samples displaying broad morphological diversity. Anastomosis (**a**), branching (**b**) and helical structures (**c**) are visible and labelled with green arrows. Scale bars = 50 μm


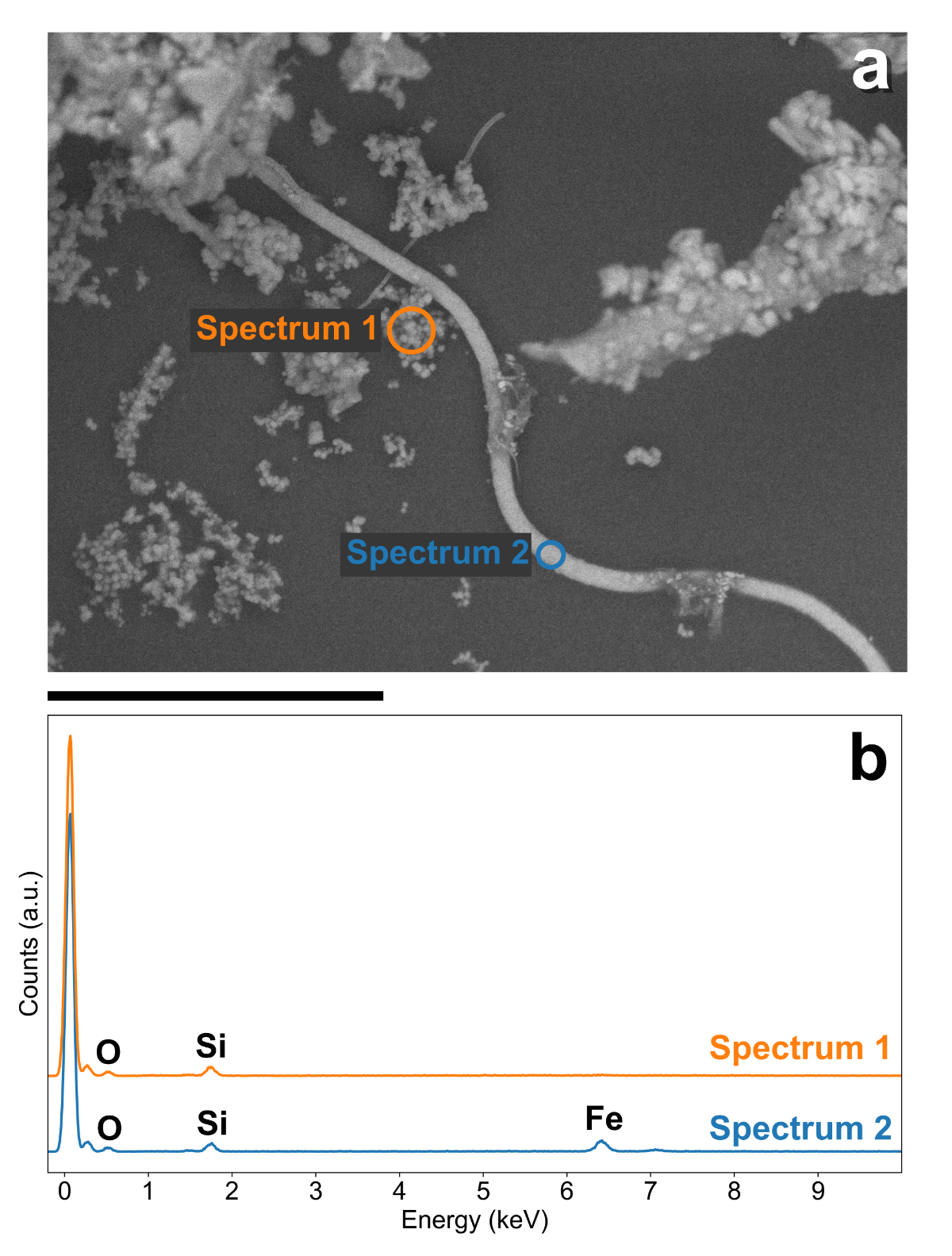


**Supplementary Figure 2:** (**a**) Photomicrograph of iron-mineralised chemical garden material artificially matured at 300°C with spectra areas labelled. Spectrum 1 (orange) was targeted at an isolated group of microspheres. Spectrum 2 (blue) was targeted at the filamentous material (**b**) EDS spectra acquired from ellipses labelled in photomicrograph with peaks labelled with associated elements. Scale bar = 50 μm


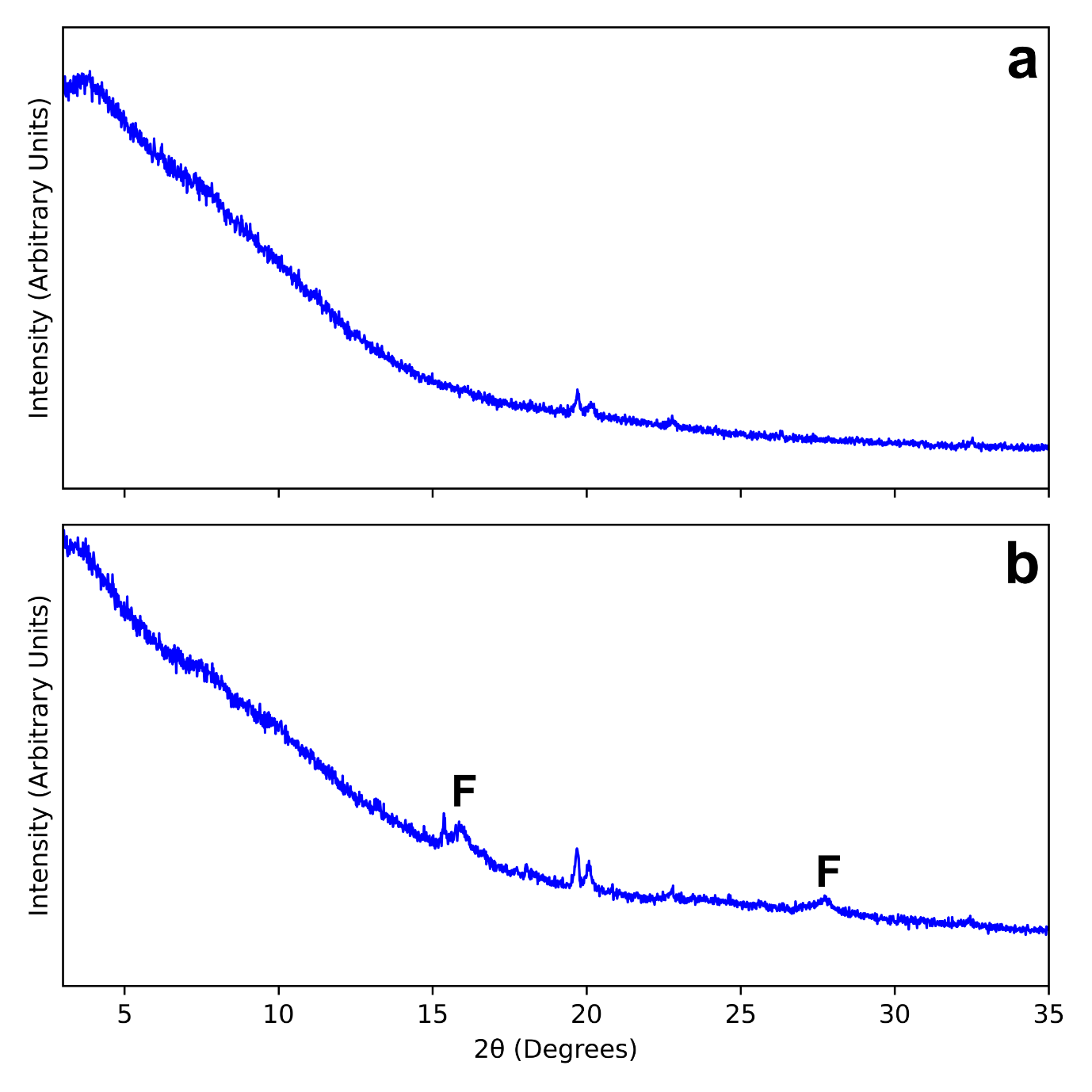


**Supplementary Figure 3:** (**a**) XRD pattern of the background signal from the foil and glue used for sample preparation. (**b**) XRD pattern of unheated iron-mineralised chemical garden sample showing the same background peaks alongside two peaks matching those of ferrihydrite in the reference database (labelled F).


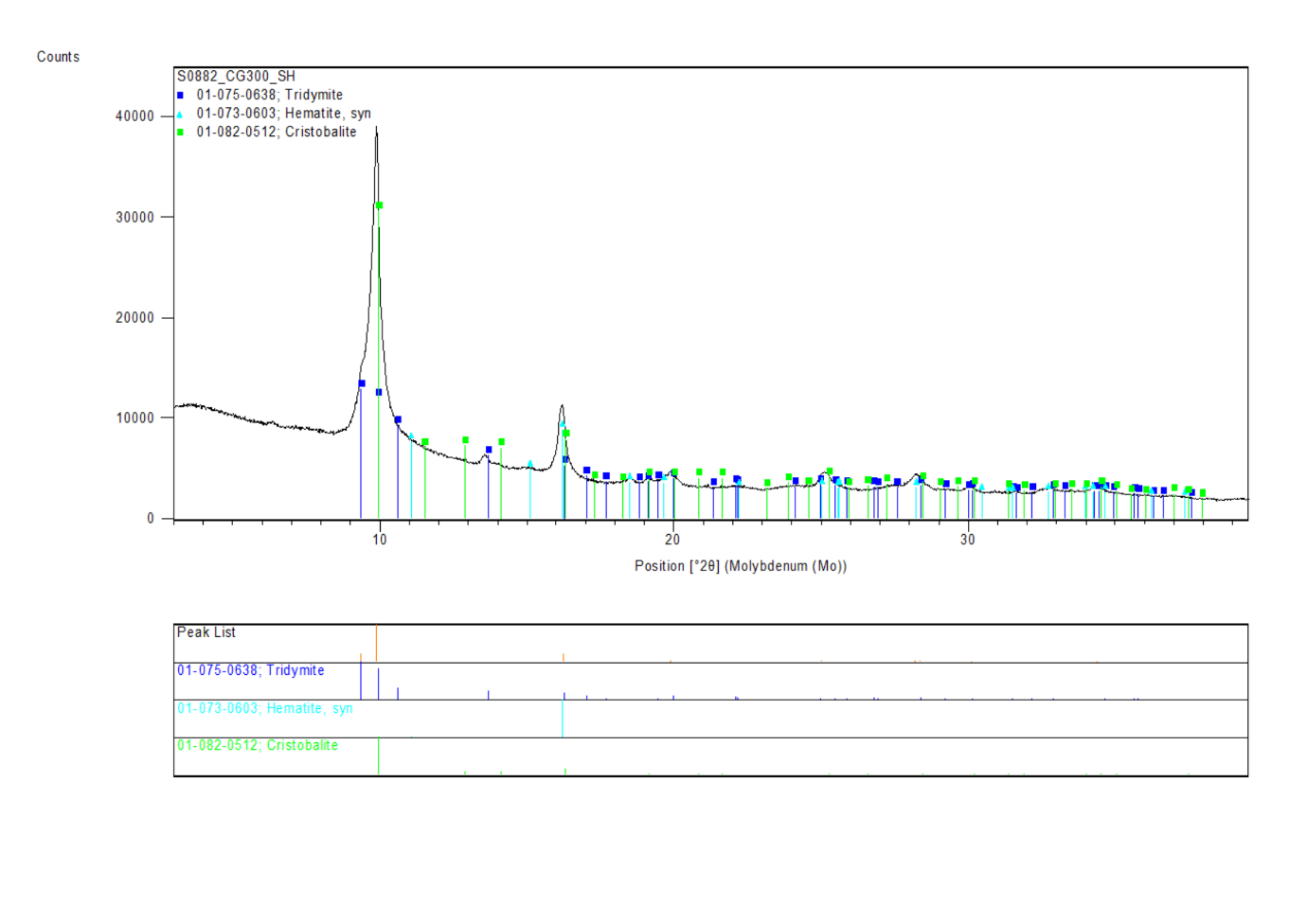


**Supplementary Figure 4:** XRD pattern of the sample artificially matured at 300°C. Reference peaks for tridymite, haematite and cristobalite used for peak assignment are shown below.
